# Evaluation of Preparation and Detoxification of Hemicellulose Hydrolysate for Improved Xylitol Production from Quinoa Straw

**DOI:** 10.3390/ijms24010516

**Published:** 2022-12-28

**Authors:** Tingwei Jin, Xiwen Xing, Yubing Xie, Yan Sun, Sijia Bian, Liying Liu, Guang Chen, Xinzhe Wang, Xiaoxiao Yu, Yingjie Su

**Affiliations:** 1 College of Life Science, Jilin Agricultural University, Changchun 130118, China; 2 Key Laboratory of Straw Comprehensive Utilization and Black Soil Conservation, The Ministry of Education, Jilin Agricultural University, Changchun 130118, China

**Keywords:** quinoa straw, detoxification, inhibitors, activated carbon, xylitol

## Abstract

Quinoa straw is rich in hemicellulose, and it could be hydrolyzed into xylose. It is a promising energy resource alternative that acts as a potential low-cost material for producing xylitol. In this study, quinoa straw was used as a substrate subjected to the hydrolysis of dilute sulfuric acid solution. Based on the production of xylose and inhibitors during hydrolysis, the optimal conditions for the hydrolysis of hemicellulose in quinoa straw were determined. Detoxification was performed via activated carbon adsorption. The optimal detoxification conditions were determined on the basis of major inhibitor concentrations in the hydrolysate. When the addition of activated carbon was 3% at 30 °C for 40 min, the removal of formic acid, acetic acid, furfural, and 5-HMF could reach 66.52%, 64.54%, 88.31%, and 89.44%, respectively. In addition to activated carbon adsorption, vacuum evaporation was further conducted to perform two-step detoxification. Subsequently, the detoxified hydrolysate was used for xylitol fermentation. The yield of xylitol reached 0.50 g/g after 96 h of fermentation by *Candida tropicalis* (CICC 1779). It is 1.2-fold higher than that obtained through the sole vacuum evaporation method. This study validated the feasibility of xylitol production from quinoa straw via a biorefinery process.

## 1. Introduction

Owing to its extensive utilization and reproducibility, lignocellulose waste is a common low-cost energy resource in nature [1]. However, it is still challenging to convert lignocellulose biomass into valuable chemicals. Further exploitation and utilization of lignocellulose resources are worth more research attention [2]. Recently, an increasing number of studies focus on the conversion of (hemi-)cellulose in lignocellulose materials into high value-added products, such as xylitol and bioethanol [3,4]. As a highly nutritive cereal, quinoa has been recommended to be the most suitable staple food for humans by FAO [5]. Quinoa production increased to 148,700 tones in 2016, which is approximately 3 times higher than that in 2008 (50,000 tones) [4]. With the increasing demand for quinoa, its annual production gradually rises. Concomitantly, it generates an increasing number of byproducts, e.g., straw and testa. Quinoa straw could be used to produce high-value products through biorefinery, considering the abundant (hemi-)cellulose that contains structural units of xylose, glucose, and arabinose [6,7].

Xylitol is a low-energy artificial sweetening agent, which could prevent tooth decay, tooth hardening, otitis media as well as ear and respiratory infections [8]. Thus, it is a sugar alternative in the food and pharmaceutical industries. Xylitol production could be achieved mainly through the chemical reduction of xylose. However, it consumes great energy and cost and causes serious pollution, considering the use of metal catalysts (e.g., Ni, Pd and Ru) during conversion. These drawbacks limit its application in the food and pharmaceutical industries [9]. On the other hand, xylitol production from the biorefinery of lignocellulose could not only enhance the added value of renewable resources but also reduce waste and environmental pollution caused by the improper use of straws [10,11,12].

The conversion of xylose to xylitol occurs mainly through the reduction of xylose to xylitol by NAD(P)H-dependent xylose reductase (XR) [13]. Currently, *Paraburkholderia sacchari*, *Burkholderia sacchari* et al. can convert xylose to produce xylitol. Among the yeasts, *Candida* is considered to be the most commonly used for the fermentation of xylitol [14,15,16]. Misra et al. studied the production capacity of 18 yeast species and *Candida tropicalis* showed good performance in the production of xylitol. This could be due to the fact that *Candida tropicalis* was found to have the higher XR activity. Some reports indicate that microorganisms with high XR activity and NADPH-dependent characteristics are likely to be potential producers of xylitol from xylose [17,18]. In addition, the tolerance and degradation rate of *Candida tropicalis* to furfural inhibitors produced by acid hydrolysis in medium were also higher than those of most wild type strains [19,20].

The first step in xylitol biorefinery using lignocellulose as the substrate is to separate and hydrolyze hemicellulose and convert it to fermentable sugars [21,22]. The most common and rapid method for hydrolyzing hemicellulose is dilute sulfuric acid (DSA) hydrolysis [23]. However, the major problem of this method is the generation of some inhibitors (e.g., formic acid, acetic acid, furfural, hydroxymethylfurfural, and phenolics) during hydrolysis. These compounds would influence cell metabolism in the subsequent fermentation process [24,25,26,27]. Therefore, detoxification prior to fermentation is necessary to reduce the negative effect. Common methods of detoxification include activated carbon adsorption, evaporation, ion-exchange resin, and so on. Among them, activated carbon adsorption is widely applied to detoxify acid hydrolysates because of its low cost and good adsorption efficacy [28,29]. Ahuja et al. used this method to detoxify the hydrolysate obtained upon dilute acid treatment and steam explosion, and they found out that furfural and phenolics were removed by 93% and 94%, respectively [30]. Gupta et al. used activated carbon as the adsorbent and performed fermentation of a corncob hydrolysate, they found out that two strains of *Candida tropicalis* could grow and yield sugars only in the detoxified hydrolysate and the xylitol yield reached 4.8 and 4.6 g/L, respectively [24].

**Figure 1:**
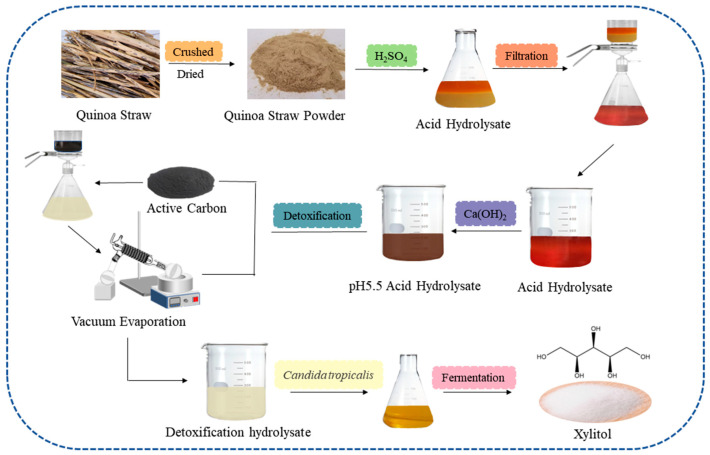
Schematic illustration of the biorefinery process for Xylitol production.

In this study, quinoa straw was used as a raw material to hydrolyze its hemicellulose into xylose through dilute acid hydrolysis. Furthermore, two-step detoxification was conducted on the hydrolysate, the optimal conditions were determined by analyzing changes in the major inhibitor concentration after detoxification and the detoxified hydrolysate was used for xylitol fermentation (Figure 1). This research could promote the conversion and utilization of quinoa straw as a renewable resource and the sustainable development of the industrial production of xylitol.

## 2. Results and Discussion

### 2.1. Chemical Composition Analysis of Quinoa Straw

Table 1 shows the chemical composition of quinoa straw and corn straw. Quinoa straw contained cellulose, hemicellulose and lignin contents of 30.95%, 20.80%, and 19.98%, respectively. In contrast, the corresponding values of corn straw were 34.25%, 17.55%, and 21.90%, respectively. The hemicellulose content in quinoa straw was 15.62% higher than that in corn straw. This made quinoa straw a more suitable material to produce xylose through saccharification.

### 2.2. Effect of the Acid-Hydrolysing Concentration and Treatment Time of Quinoa Straw on the Production of Sugars and Inhibitors

Hydrolyzing the substrate could remove lignin and reduce crystallinity, which is a key step for the subsequent saccharification of biomass and conversion into xylitol [8]. DSA hydrolysis is a common method to depolymerize hemicellulose in lignocellulose biomass into sugar monomers. It has extensive applications due to its low cost and non-volatile characteristics [31]. DSA treatment of lignocellulose in a certain temperature range promotes the exposure of cellulose and liquefaction of hemicellulose, thus resulting in highly digestible cellulose in the solid residue and hemicellulose monomer in the liquid part [32]. The treatment conditions of acid hydrolysis were optimized to obtain hydrolysates with a higher sugar content. Nevertheless, dilute acid hydrolysis would generate inhibitors that interfere with the growth and metabolism of microbes. Therefore, diminishing the influence of inhibitors is also an indispensable factor during optimization.

The hydrolysis rate and efficiency could be affected by the acid concentration and reaction time [33]. Hence, these parameters are optimized to maximize the yield of sugars.

**Figure 2:**
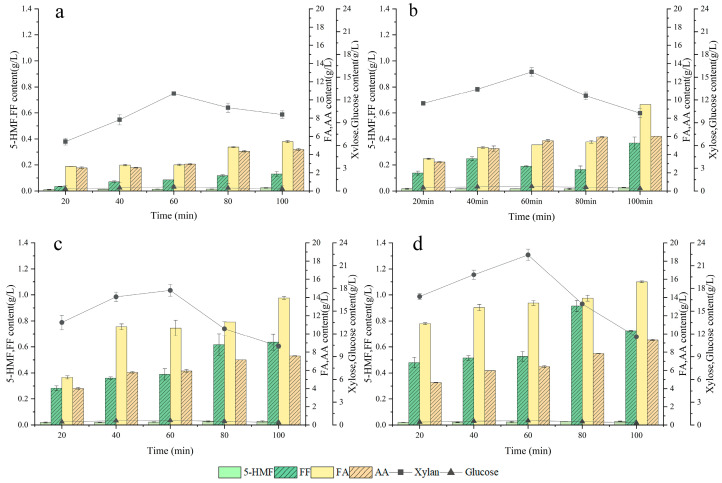
Effect of DSA concentration ((**a**): 1%, (**b**): 2%, (**c**): 3% and (**d**): 4%) and treatment time on the production of sugars and inhibitors (FA: formic acid; AA: acetic acid; 5-HMF: 5-hydroxymethylfurfural; FF: furfural).

The main components in the hydrolysate were detected by HPLC (Figures S2 and S4). The contents of sugars and inhibitors were obtained through the calculation of the peak area (Figure 2). Within the DSA concentration range of 1–4%, no significant difference was observed in the glucose concentration (below 1 g/L). However, the xylose concentration significantly increased with the increment of DSA concentration, which reached its peak (over 15 g/L) at a DSA concentration of 2%. It was clear that the xylose concentration in the quinoa straw hydrolysate was markedly higher than the glucose concentration. The glycosidic bonds in hemicellulose would be broken down and generate xylooligosaccharides and xylose. Moreover, hemicellulose suffers from significant depolymerization with the enhanced treatment severity, along with which the xylose concentration gradually increases. The lower glucose concentration might be explained by less degradation of cellulose under these conditions, and the glucose in the hydrolysate is mainly derived from side chains of hemicellulose [34]. In this line, Yu et al. proposed that the release of pentose from hemicellulose is easier than that of hexose [35]. Despite the lower concentration of glucose in the hydrolysate, it could avoid the formation of ethanol via anaerobic respiration that inhibits xylitol production [36]. Additionally, both glucose and xylose concentrations increased with the prolonged treatment time and reached their peak after 60 min. When 4% DSA was used for 60 min, the xylose concentration in the hydrolysate reached a maximum (20.42 g/L). When the treatment time exceeded 60 min, the xylose concentration tended to decrease with time. It was mainly attributed to the generation of furfural via decomposition of xylose, which may polymerize with xylose, namely the generation rates of both xylose and byproducts increased as the reaction proceeded; secondary reactions were faster than the intended reactions after certain treatment durations [37].

The contents of the four inhibitors had positive correlations with treatment intensity (time and DSA concentration). When a lower DSA concentration was used, the increase in the inhibitor content was very slow (Figure 2a). When the DSA concentration exceeded 3%, the inhibitor content increased (Figure 2c) and the formic acid, acetic acid, furfural and 5-HMF contents were above 5.23 g/L, 4.01 g/L, 0.28 g/L and 0.02 g/L, respectively. The contents of the four inhibitors increased dramatically after 60 min. This was also one of the reasons why xylose and glucose concentrations decreased after 60 min of treatment. After hydrolyzing with 4% DSA for 100 min, contents of formic acid, acetic acid, furfural, and 5-HMF reached their highest values, which were 13.94 g/L, 7.58 g/L, 0.64 g/L, and 0.03 g/L, respectively. The increase in the inhibitor content was related to the rupture of glycosidic bonds by DSA. Nevertheless, the rupture did not selectively occur on cellulose, hemicellulose, glucose, or xylose. This weak selectivity causes glucose and xylose to be excessively degraded into furfural and formic acid [38]. Acetic acid is released from the hydrolysis of acetylated hemicellulose [39]. The increase in the acetic acid content indicated great hydrolysis of hemicellulose. Clearly in the figures, the formic acid and acetic acid contents were markedly higher than those of furan-like inhibitors. The lower furfural content might be attributed to the fact that it could react with degradation products of pentose under the catalysis of DSA, which could further form humus that was converted to gas [40]. Additionally, pentose (xylose and arabinose) could partially degrade into furfural, while hexose (glucose, galactose and mannose) could degrade into 5-HMF. In this study, the xylose concentration in the hydrolysate was markedly greater than that of glucose, which coincided with the higher level of furfural than that of 5-HMF. In summary, 3% DSA treatment for 60 min was selected as the optimal hydrolysis condition, at which lower inhibitor contents were obtained to reduce their influence on fermentation.

### 2.3. Effect of Hydrolysis Temperature on the Production of Sugars and Inhibitors

**Figure 3:**
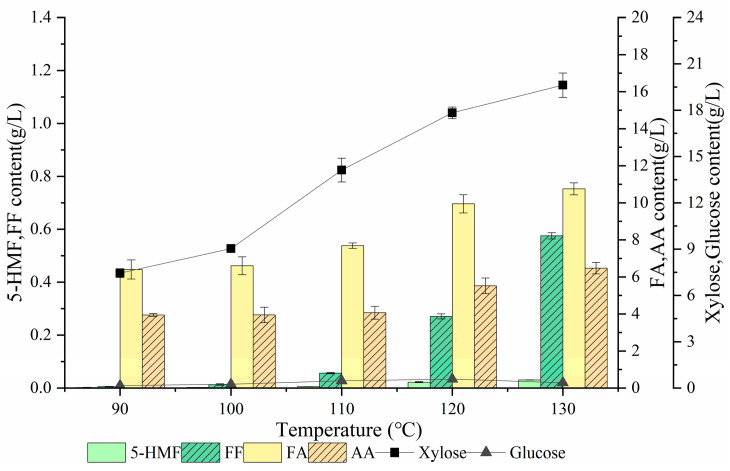
Effect of treatment temperature on the production of sugars and inhibitors. (FA: formic acid; AA: acetic acid; 5-HMF: 5-hydroxymethylfurfural; FF: furfural).

A proper treatment temperature is requisite for producing sugars. Treatment temperatures were optimized after obtaining the DSA concentration and treatment time. As shown in Figure 3, both the xylose and glucose contents increased as the treatment temperature increased. At 120 °C, the glucose content reached the highest level (0.58 g/L); subsequently, it decreased to 0.34 g/L. The hydrolysis of cellulose was not obvious when the temperature was below 120 °C, and glucose was mainly derived from heteropolymers of the hemicellulosic fraction [41]. When the temperature exceeded 120 °C, the glucose content decreased probably because of its conversion to 5-HMF. At 90 °C, the xylose content was 7.45 g/L, and it gradually increased with the rising temperature. At 130 °C, it increased to 19.62 g/L. The temperature increase induced softening of the lignin layer surrounding hemicellulose, which made the hydrolysis of cellulose into xylose easier [42]. Nevertheless, hemicellulose and cellulose would depolymerize at higher temperatures and generate more inhibitors [43]. Among the major degraded byproducts, the formic acid content increased from 6.40 g/L to 10.75 g/L, while the acetic acid content increased from 3.94 g/L to 6.47 g/L. The lower hexose content in the hydrolysate indicated the lower level of 5-HMF, and the maximum of the latter was only 0.03 g/L. In contrast, the maximum of furfural reached 0.57 g/L. Based on the effect of temperature on the production of sugars and inhibitors, 120 °C was selected as the optimal treatment temperature, the xylose content was 17.83 g/L.

### 2.4. Effect of Activated Carbon Addition on the Detoxification of Quinoa Straw Hydrolysates

The lignocellulose hydrolysate should be usually detoxified prior to fermentation because the presence of inhibitors may be toxic to microorganisms and inhibit their metabolism, and even significantly inhibit the production of xylitol [25,44]. Activated carbon adsorption could effectively remove most inhibitors, but it could lead to sugar loss because some sugars are adsorbed along with the absorption of inhibitors. Therefore, sugar loss is an important measure of adsorption efficiency [45]. Colored substances (pigments) in the hydrolysate are thought to be impurities generated from the oxidation of phenolic compounds during hydrolysis. The removal of this type of impurity is also a vital index for the activated carbon adsorption method. Thus, the decolorization rate could act as another indicator of detoxification efficiency [46,47].

**Figure 4:**
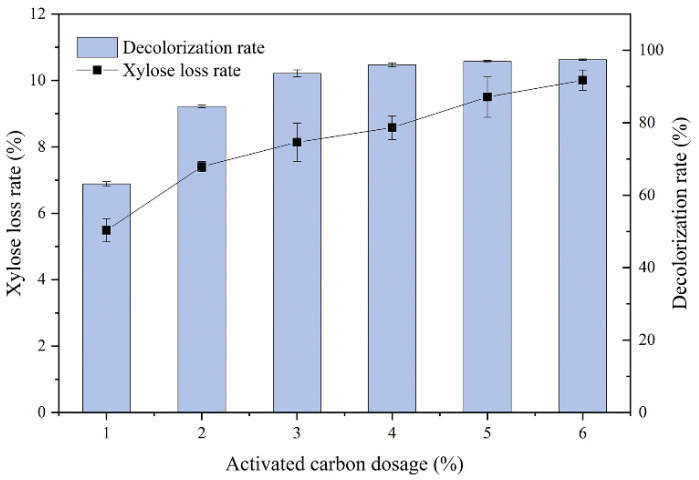
Effect of the activated carbon dosage on the decolorization rate and sugar loss.

Figure 4 depicts the effect of activated carbon addition on the sugar loss and decolorization rate. Its addition level ranged from 1% to 6%, and detoxification was performed at 30 °C for 10 min. The decolorization rate significantly increased with an enhanced addition level of activated carbon. It increased from 63.11% (the initial value) to 93.64% when the addition level was 3%. Subsequently, the increasing trend became steady. The decolorization rate reached the highest (97.43%) when the addition level was 6%. The detoxification process depends on the pore size and surface functional groups. Thus, a moderate increase in activated carbon addition is favorable for adsorption [48]. However, excessive amounts of activated carbon would result in xylose loss. The addition level of 6% increased the sugar loss to 10%, which was 18.68% greater than that of the 3% addition level. Therefore, the addition level of 3% was selected, considering its higher decolorization rate and lower xylose loss. Similarly, Kumar et al. found out that the 2.5% addition level of activated carbon and treatment for 40 min resulted in a decolorization rate of up to 95% with a xylose loss of 8%. Moreover, they concluded that the xylose loss reached up to 15%, as the addition level of activated carbon increased [47].

### 2.5. Effect of Temperature and Duration of Activated Carbon Adsorption on Detoxification Efficiency

Byproducts generated during DSA treatment in the hydrolysate may inhibit fermentation, such as formic acid, acetic acid, furfural, and 5-HMF. Thus, detoxifying the hydrolysate via adsorption is necessary for the subsequent xylitol fermentation. The adsorption efficiency of activated carbon is determined by several parameters, such as adsorption time, temperature, and activated carbon concentration [30].

**Figure 5:**
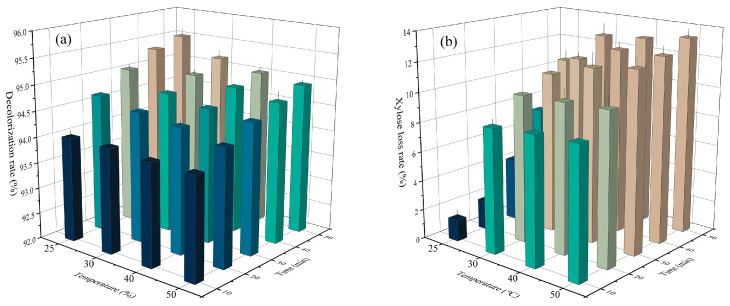
Effect of adsorption temperature and time on the decolorization rate (**a**) and sugar loss (**b**).

Clearly from Figure 5a, a decolorization rate of 94% was achieved when detoxification was performed at 25 °C for 10 min. As the adsorption time was prolonged, the decolorization rate increased and reached 95.5% after 50 min of treatment. However, it gradually decreased as the treatment temperature increased, and it decreased to 93.9% upon treating at 50 °C for 10 min. Hence, the activated carbon showed weaker adsorption affinity for pigments at higher temperatures, which made it not suitable for high-temperature adsorption.

Significant changes were also observed in sugar loss, as the treatment temperature varied (Figure 5b). It reached 13.35% after treating at 50 °C for 50 min, which was increased by 21.27% in contrast to that at 25 °C. In addition, the activated carbon showed a similar sugar adsorption efficiency at a temperature of 30–50 °C, at which the sugar loss was approximately 13%. As inferred by Lee et al., sugars were not chemically attached to activated carbon, but they might be entrapped by some physical interactions [48]. Concomitantly, Ahuja V.et al. mentioned that the adsorption of activated carbon depended on its pores, which are little accessible for macromolecules like carbohydrates. Thus, strong bonds and chemical adsorption are required for their interactions [28].

**Figure 6:**
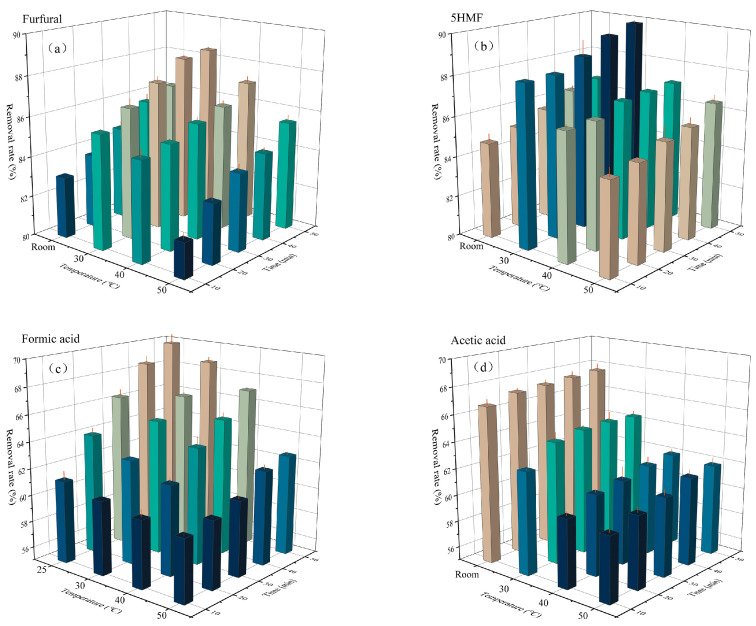
Effect of adsorption temperature and time on removal of furfural (**a**), 5-HMF (**b**), formic acid (**c**), and acetic acid (**d**).

Activated carbon adsorption was conducted at 25 °C, 30 °C, 40 °C, and 50 °C with its addition level of 3%. As illustrated in Figure 6, activated carbon presented a certain adsorption efficiency for inhibitors. As the adsorption time was prolonged, the adsorption efficiency improved and the removal of inhibitors became higher. However, the adsorption efficiency was negatively correlated with the treatment temperature. A higher temperature resulted in a lower adsorption efficiency. The likely cause was that the reaction released heat and induced an increase in temperature, but activated carbon exhibits better adsorption at low temperatures. At a treatment temperature of 30 °C 50 min, the removal of furfural and 5-HMF reached 88.45% and 88.92%, respectively. As the temperature increased, their removal declined, i.e., the removal at 50 °C (only 85.47% and 86.46%, respectively) was much lower than that at 30 °C (Figure 6a,b). Further, Gupta et al. mentioned that the removal of furan was dependent on the temperature, and the best removal efficiency was achieved at 30 °C [24]. The physical adsorption efficiency will become better if the treatment time gets prolonged. The adsorption efficiency of activated carbon depends on its pores, which are accessible for smaller molecules like furfural. As the adsorption time prolongs, pores may be blocked so the adsorption efficiency declines [48,49]. In this study, therefore, the removal increase became slower after 40 min of detoxification, which is probably attributed to the saturation of activated carbon.

Compared with inhibitors like furfural, acids had presented a weaker removal efficiency (approximately 60%). For inhibitors like formic acid and acetic acid, their adsorption efficiency also significantly decreased with the increasing temperature (Figure 6c,d). Their removal efficiency reached 69.79% and 67.61%, respectively, after treating at 25 °C for 50 min. However, upon treatment at 50 °C for 50 min, their removal efficiency declined by 10.28% and 8.53%, respectively. Similar findings were achieved by Soleimani et al., who conducted activated carbon adsorption and found out that the adsorption efficiency of acetic acid was much lower than that of phenol and furfural [46]. Further, Lee et al. found out that the removal of furans was better than that of acids, and the adsorption of 5-HMF and furfural could reach about 100% after 1 h of treatment. However, in their study, the adsorption of formic acid and acetic acid was much lower [48]. Presumably, furans have a stronger structural affinity to activated carbon than acids. To maximize the removal of inhibitors and the decolorization rate and reduce sugar loss, treatment at 30 °C for 40 min was selected for detoxification in the following process, the removal of formic acid, acetic acid, furfural, and 5-HMF were 66.52%, 64.54%, 88.31%, and 89.44%.

### 2.6. Effect of Detoxification Methods on Xylitol Fermentation

The effect of different detoxification methods on xylitol yield was evaluated to enhance the subsequent fermentation performance of the quinoa straw hydrolysate. We prepared detoxified hydrolysates obtained from vacuum evaporation and from activated carbon adsorption–vacuum evaporation (two-step method) with equivalent xylose concentration, which were subjected to analysis of the inhibitor content. After fermentation by *Candida tropicalis CICC 1779*, fermented aliquots were collected for xylitol content analysis (Figure S5).

Table 2 presents the content of the four inhibitors in the hydrolysate with or without detoxification treatments. The hydrolysate was concentrated by about 3.3-fold when vacuum evaporation was used; concomitantly, all inhibitor contents were significantly changed. The removal of furans was more obvious, and especially, furfural was removed by 92.31% (decreased from the initial 0.26 g/L to 0.02 g/L). Villarreal et al. removed the amount of furfural by 97% through evaporation. A study also reported that the reduction of furfural might be related to its polymerization at high temperatures [50]. However, regarding acids, their concentration got slightly increased due to concentration. In addition, Zhang et al. found out that vacuum evaporation resulted in a weaker removal efficiency of acetic acid, while it was more suitable for sugar concentration and removal of furan inhibitors [51]. Regarding the two-step method (activated carbon detoxification–vacuum evaporation), it showed a more pronounced efficiency for inhibitor removal. The formic acid and acetic acid contents in the detoxified hydrolysate were 8.47 g/L and 4.08 g/L, respectively. The two values were decreased by 65.43% and 64.89%, respectively, in comparison with those obtained from the sole vacuum evaporation method. Inhibitors of acids influence fermentation because they could lower the pH of the cellular environment and cause cell death [52]. With the two-step method, furfural and 5-HMF contents were close to zero. Based on the above results, the two-step method (activated carbon absorption–vacuum evaporation) induced the best removal efficiency of inhibitors in the hydrolysate.

**Figure 7:**
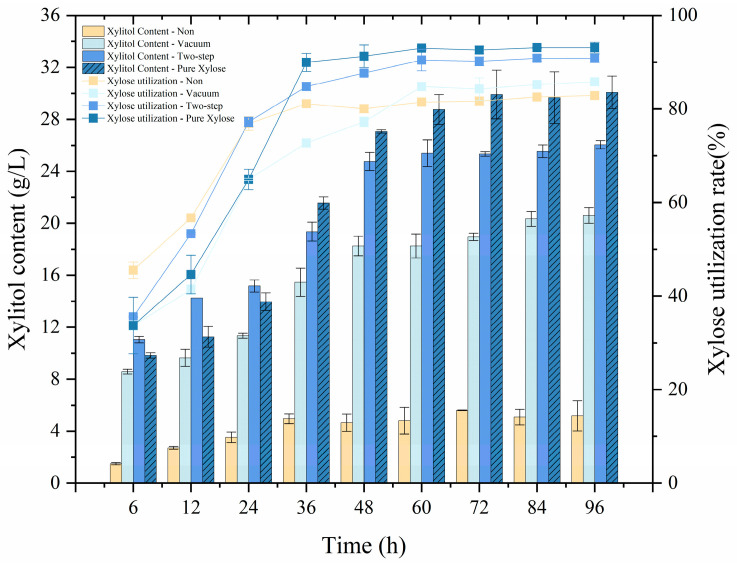
Xylitol content and xylose utilization rate obtained during fermentation.

Figure 7 shows xylitol content and xylose utilization rate obtained during fermentation of hydrolysate to xylitol. When the fermentation was performed with the two-step detoxified hydrolysate fermentation broth, the xylose utilization rate reached 77% at 24 h, and the xylitol content reached 25.40 g/L at 60 h, slightly lower than that of pure xylose fermentation medium. Table 3 reflects the specific xylitol yields and xylose utilization rates in the fermented hydrolysate with or without detoxification. As for the non-detoxified quinoa straw hydrolysate, the xylitol content was 5.57 g/L, and xylose consumption was 92.87%. However, the xylitol yield was only 0.34 g/g. Presumably, xylose was mainly utilized by microbes for metabolism, while the remnant xylose was not sufficient for xylitol fermentation. In this line, Misra et al. found out that a lower substrate concentration limited the fermentation yield and production rate [1]. A 58.56 g/L xylose concentration was obtained in the hydrolysate concentrated by vacuum evaporation; the xylitol content was 20.60 g/L after *Candida tropicalis* fermentation, and the yield reached 0.41 g/g. For the fermented hydrolysate obtained using the two-step method, the xylose consumption was 90.82%, while the xylitol content increased to 26.05 g/L with a yield of 0.50 g/g. The yield was 1.2-fold higher than that from sole vacuum evaporation. In contrast, the xylitol content was 30.08 g/L with a yield of 0.54 g/g in the pure xylose medium. This value was close to that obtained using the two-step method. This suggested that detoxification effectively reduced the inhibitor content in the hydrolysate, thus alleviating the adverse impact on xylitol fermentation. Vallejos et al. removed the inhibitors in the hemicellulose hydrolysate via a series of detoxification methods (e.g., activated carbon and ion-exchange resin), increasing the xylitol yield from 0 to 0.46 g/g. They also suggested that detoxification markedly improved xylitol yield [12]. Our results demonstrated that activated carbon–vacuum evaporation (a two-step method) could act as an effective detoxification approach to xylitol fermentation. Further, it validated the feasibility of xylitol production via biorefinery of raw quinoa straw.

## 3. Materials and Methods

### 3.1. Materials

The quinoa straw (dried and comminuted into powder to 60 mesh with a grinder, and dried at 80 °C for 12 h to dry) was obtained from the quinoa plantation in Jilin Agricultural University, Jilin Province. Activated carbon was purchased from Macklin (200 mesh). furfural, 5-HMF, formic acid, acetic acid, and xylitol standards were purchased from Beijing Chemical Reagent Co. Ltd.

### 3.2. Strain and Culture Medium

*Candida tropicalis* strain CICC.1779 was obtained from China Industrial Microbial Strain Conservation Center and stored at 4 °C on strain preservation medium.

Seed medium: D-xylose 20 g/L, yeast extract 10 g/L, peptone 20 g/L, pH 5.5. 250 mL flask containing 100 mL seed medium was incubated overnight at 30 °C, 160 rpm with 1% (*w*/*v*) inoculum.

Fermentation medium: D-xylose 60 g/L, yeast extract 5 g/L, tryptone 4 g/L. The inoculated seed solution was inoculated with 2% (*w*/*v*) inoculum into the fermentation medium and incubated at 30 °C, 160 rpm for 48 h.

### 3.3. Acid Hydrolysis

1 g quinoa straw was mixed with different concentrations of DSA (1–4%) in a 50 mL flask at a solid-liquid ratio of 1:10 (*w*/*v*) and hydrolyzed in an autoclave at different temperatures (90–130 °C) for specific durations (20–100 min). The liquid fraction was collected by filtration and the unhydrolyzed solid residue was removed.

### 3.4. Detoxification of Acid Hydrolysis

#### 3.4.1. Activated Carbon Detoxification

The quinoa straw hydrolysate was adjusted to pH 5.5 using Ca (OH)_2_ and filtered for precipitate removal. The pH adjustment procedure was followed by adsorption treatment with different dosage activated carbon (1–6% (*w*/*v*)). The reaction mixtures were incubated at different temperatures (25–50 °C), 160 rpm, for 10–50 min. The absorbance of the detoxicated hydrolysate after the removal of activated carbon will be measured at 420 nm to calculate the decolorization rate. The concentration of xylose and harmful inhibitor was determined to calculate the xylose loss rate and harmful inhibitor removal rate.

#### 3.4.2. Vacuum Evaporation Detoxification

The concentration process of hydrolysate by vacuum evaporation could further remove harmful inhibitors. The obtained hydrolysate was concentrated by vacuum evaporation at 75 °C until the xylose content was about 60 g/L. The hydrolysate was recovered for subsequent fermentation.

### 3.5. Xylitol Fermentation

The detoxified hydrolysate or pure xylose (xylose concentration of 60 g/L), supplemented with yeast extract 5 g/L, tryptone 4 g/L, pH5.5 was used as the fermentation medium. Then, 2% (*v*/*v*) seed medium was inoculated into the mixture directly after sterilization. The fermentations were performed at 30 °C and 160 rpm in the incubator shaker for 48 h. The experiment was carried out in triplicate.

### 3.6. Composition Analysis of Corn Straw

The straw was crushed and dried in an oven and treated according to the NREL method of the National Renewable Energy Laboratory, using a two-step sulphuric acid hydrolysis to completely hydrolyse the cellulose and hemicellulose to monosaccharides [53]. Lignin was quantified by cauterisation at 575 °C in a muffle furnace. Corn straw and quinoa straw were determined separately and each determination was carried out in triplicate [54].

### 3.7. Analysis Methods

Inhibitor components were analyzed by HPLC (Waters 1525) equipped with a UV detector (Waters 2489) and a SilGreen ODS C18 column. Furfural and 5-HMF were measured at 280 nm with a flow rate of 1 mL/min, and methanol/water (1:9, *v*/*v*) as mobile phase. Formic acid and acetic acid were measured at 210 nm with a flow rate of 0.7 mL/min, KH_2_PO_4_/methanol (2:8, *v*/*v*) as mobile phase (Figure S1).

The content of xylitol, xylose, and glucose was determined using a determined using refractive index detector with an Inertsil Sugarpak1 (6.5 mmid × 300 mm) column and ultra-pure water as the mobile phase. The flow rate was 0.4 mL/min and the detection was carried out at a column temperature of 85 °C. All mobile phases and samples were filtered through a 0.22 μm filter and used in the HPLC analysis (Figure S3). Xylitol yield is calculated by the following equation, calculated according to Equation (1):

(1) $$\mathrm{The}\;\mathrm{yield}\;\mathrm{of}\;\mathrm{xylitol}\;(g/g)=\frac{\mathrm{The}\;\mathrm{concentration}\;\mathrm{of}\;\mathrm{xylitol}}{\mathrm{Intial}\;\mathrm{xylose}\;\mathrm{concentration}-\mathrm{Residual}\;\mathrm{xylose}\;\mathrm{concentration}}$$

## 4. Conclusions

Based on quinoa straw as the raw material, we produced xylitol through a biorefinery process. After the optimization of DSA hydrolysis parameters, the xylose concentration in the hydrolysate reached 17.83 g/L. Activated carbon adsorption was performed for detoxification to obtain the hydrolysate. The optimal detoxification conditions were determined through analyzing changes of the major inhibitor content in the hydrolysate after detoxification. Under optimal conditions, the removal of formic acid, acetic acid, furfural and 5-HMF reached 66.52%, 64.54%, 88.31%, and 89.44%, respectively. Further, the quinoa straw hydrolysate was detoxified via activated carbon adsorption–vacuum evaporation (a two-step method), and the detoxified hydrolysate was used for xylitol fermentation. It resulted in a xylitol yield of 0.50 g/g. Our results demonstrated that DSA treatment conditions could affect the fermentation of the hydrolysate by modulating its fermentable sugars and inhibitor content. The two-step method enhanced xylitol yield by removing toxic compounds, which is also one of the critical steps during a biorefinery process. As a lignocellulose biomass, the quinoa straw hydrolysate is a potential alternative material for xylitol production.

## Figures and Tables

**Table 1:** Chemical compositions of quinoa straw and corn straw.

Straw	Cellulose%	Hemicellulose%	Lignin%
Quinoa straw	30.95 ± 0.48	20.80 ± 0.64	19.98 ± 0.55
Corn straw	34.25 ± 0.49	17.55 ± 0.44	21.90 ± 0.22

**Table 2:** Formic acid, acetic acid, 5-HMF and furfural contents in the hydrolysate with or without detoxification treatment.

**Treatment**	**Formic Acid** **(g/L)**	**Acetic Acid** **(g/L)**	**5-HMF** **(g/L)**	**Furfural** **(g/L)**
Non-detoxified hydrolysate	9.53 ± 0.57	5.02 ± 0.05	0.03 ± 0.00	0.26 ± 0.00
Vacuum evaporation	24.50 ± 0.47	11.62 ± 0.47	0.04 ± 0.00	0.02 ± 0.00
Activated carbon- vacuum evaporation detoxification	8.47 ± 0.65	4.08 ± 0.16	0.01 ± 0.00	0

**Table 3:** Effects of detoxification treatment on the production of xylitol.

Treatment	Xylose	Xylose Utilization	Xylitol Production		
	(g/L)	(%)	Concentration (g/L)	Productivity (g/L/h)	Yield (g/g)
Non-detoxified hydrolysate	17.82 ± 0.23	82.87 ± 0.42	5.57 ± 1.16	0.06 ± 0.00	0.34 ± 0.00
Vacuum evaporation	58.56 ± 0.15	85.82 ± 0.66	20.60 ± 0.61	0.21 ± 0.01	0.41 ± 0.01
Activated carbon-vacuum evaporation	57.37 ± 0.56	90.82 ± 0.85	26.05 ± 0.31	0.27 ± 0.01	0.50 ± 0.01
Pure Xylose	60.00 ± 0.00	93.08 ± 1.15	30.08 ± 1.25	0.31 ± 0.01	0.54 ± 0.01

## Data Availability

All data generated or analyzed during this study are included in this published article and its additional files.

## Supplementary Materials

The following supporting information can be downloaded at: https://www.mdpi.com/article/10.3390/ijms24010516/s1.
