# Supplementary material for: Evaluation of Preparation and Detoxification of Hemicellulose Hydrolysate for Improved Xylitol Production from Quinoa Straw
Source: Int J Mol Sci. 2022 Dec 28;24(1):516. doi: 10.3390/ijms24010516 (PMC9820623; doi:10.3390/ijms24010516)
Supplement: Supplementary file 1 [file ijms-24-00516-s001.zip › Supplementary Material.pdf]

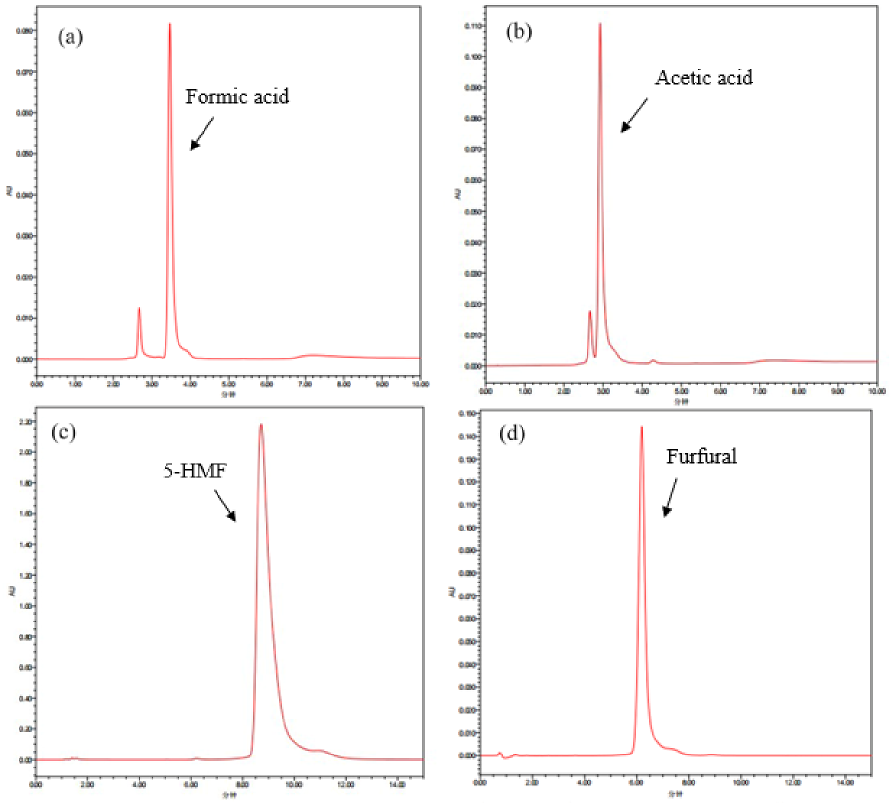

Figure S1. HPLC chromatogram of standard (a) Formic acid standard (2.9min), (b) Acetic acid standard (3.5min), (c) 5-HMF standard (6.2min), (d) Furfural standard (8.8min)。.

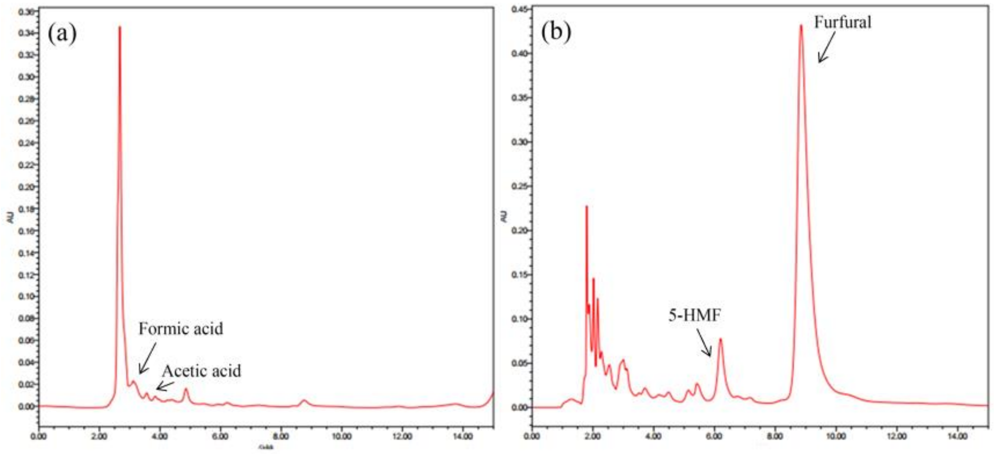

Figure S2. HPLC chromatogram of inhibitors produced during the DSA hydrolysis of quinoa straw (a) Formic acid (2.9min) and acetic acid (3.5min); (b) 5-HMF (6.2min) and Furfural (8.8min).

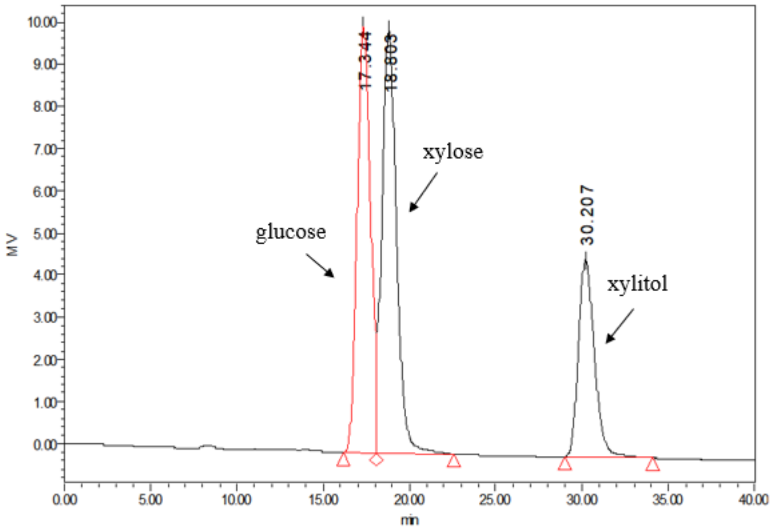

Figure S3. HPLC chromatogram of glucose (17.3min), xylose (18.8min) and xylitol (30.2min) standards.

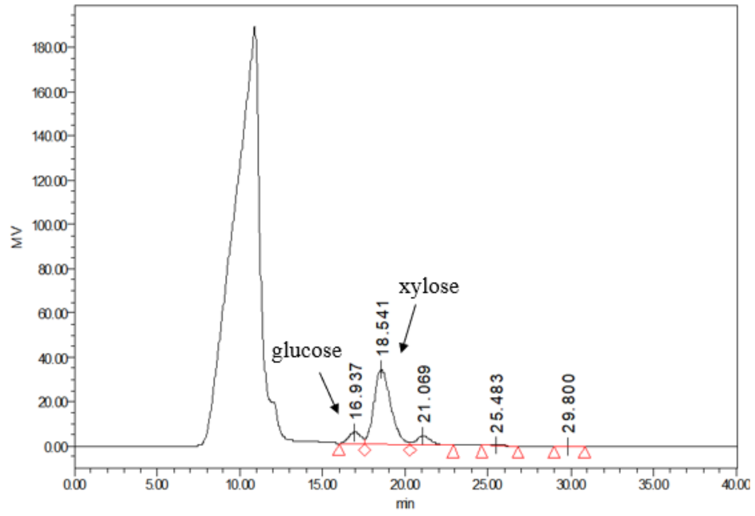

Figure S4. HPLC chromatogram of glucose (16.9min) and xylose (18.5min) produced during the DSA hydrolysis of quinoa straw.

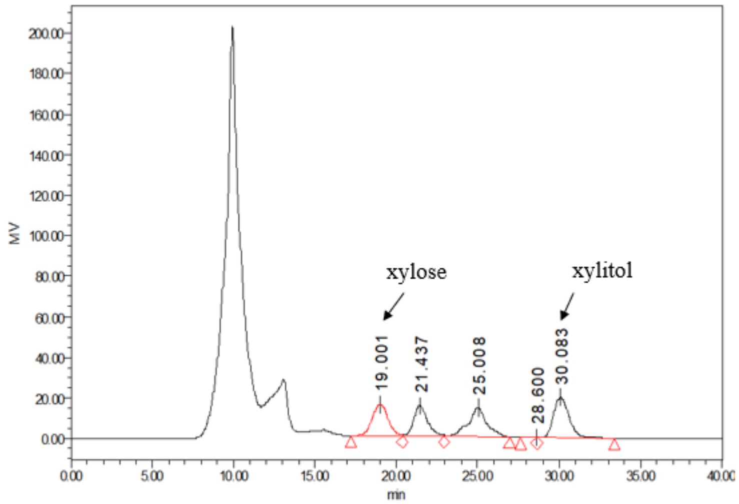

Figure S5. HPLC chromatogram of xylose (19min) and xylitol (30.1min) produced during the fermentation.
